# Role of Computed Tomography Findings in Diagnosing Pulmonary Atresia With Intact Ventricular Septum and Right Ventricle-Dependent Coronary Circulation: A Case Report

**DOI:** 10.7759/cureus.83914

**Published:** 2025-05-11

**Authors:** Kyle E Thurmann, Randy R Richardson

**Affiliations:** 1 Department of Radiology, Creighton University School of Medicine, St. Joseph's Hospital and Medical Center, Phoenix, USA

**Keywords:** case report, computed tomography imaging, congenital heart disease, patent ductus arteriosus, pulmonary atresia, right ventricular coronary circulation

## Abstract

We present a case of pulmonary atresia with intact ventricular septum and suspected right ventricle-dependent coronary circulation in a full-term neonate, highlighting the diagnostic contribution of computed tomography (CT) in evaluating a rare constellation of cardiac and airway anomalies. CT imaging demonstrated markedly dilated coronary arteries, right ventricle hypertrophy and hypoplasia, a tortuous patent ductus arteriosus causing airway compression, and complete pulmonary atresia. This complex anatomy significantly influenced the surgical decision-making process. The case illustrates CT’s role in delineating both intracardiac and extracardiac anatomy relevant to perioperative planning.

## Introduction

Pulmonary atresia with intact ventricular septum (PA/IVS) is a rare and severe congenital heart defect, occurring in approximately four to eight per 100,000 live births [1]. The subset of patients with right ventricle-dependent coronary circulation poses increased risks during surgical intervention due to the possibility of myocardial ischemia when right ventricular pressure is altered [2]. Accurate preoperative imaging is essential to evaluate the extent of coronary artery involvement and to assess the anatomy of the right ventricle, pulmonary arteries, and associated vascular structures [3].

While echocardiography remains the first-line imaging modality, its limitations in evaluating extracardiac structures have led to increased use of computed tomography (CT) and magnetic resonance imaging (MRI) in congenital heart disease. CT offers superior spatial resolution (~0.5 mm) compared to MRI (~1.5-2 mm), making it ideal for providing a detailed visualization of cardiac anatomy [4]. Recent advances have also introduced 3D CT modeling, which allows for a better understanding of the complex spatial relationships of cardiac structures for virtual surgical planning and optimization of outcomes [5]. Importantly, CT uniquely provides a detailed evaluation of airway compression by vascular structures, a critical but under-recognized complication in congenital heart disease [6].

Although each of these anomalies has been described in isolation, the convergence of right ventricle hypertrophy and hypoplasia, extensive coronary sinusoids, and airway, compromising ductal tortuosity, creates a uniquely complex clinical scenario. We describe the clinical presentation, echocardiographic findings, and CT imaging features of a neonate with PA/IVS and multiple coronary sinusoids, in whom CT helped delineate cardiovascular and airway anatomy and supported the multidisciplinary decision for single-ventricle palliation.

## Case presentation

A male neonate was born at 39 weeks gestation via cesarean section for non-reassuring fetal heart tones. Birth weight was 3.365 kg with Apgar scores of eight and nine at one and five minutes, respectively. Shortly after birth, the patient developed significant cyanosis with markedly decreased oxygen saturation despite supplemental oxygen, prompting transfer to the NICU. An initial cardiac screening ultrasound suggested ductal-dependent congenital heart disease, and prostaglandin E therapy was started to maintain ductal patency.

Physical exam at this time revealed a 3/6 systolic and diastolic murmur at the left sternal border, cyanosis of the extremities, and otherwise normal systemic findings. Pulse oximetry prior to intubation demonstrated decreased oxygen saturations, while laboratory testing revealed an elevated white blood cell count with normal hemoglobin and hematocrit (Table 1). Due to persistent hypoxemia, the patient was subsequently intubated. Arterial blood gas values obtained after intubation reflected improved oxygenation compared to pre-intubation pulse oximetry (Table 2).

**Table 1:** Selected laboratory and pulse oximetry findings prior to intubation

Laboratory Test	Observed Value	Reference Range	Interpretation
Hemoglobin	16.9 g/dL	13.5-17.5 g/dL	Normal
Hematocrit	48.9%	39-52%	Normal
White blood cell count	23.5×10⁹/L	4.0-11.0×10⁹/L	Elevated
Oxygen saturation (pulse oximetry) – Preductal	68%	>95%	Decreased
Oxygen saturation (pulse oximetry) – Postductal	66%	>95%	Decreased

**Table 2:** Arterial blood gas values following intubation

Laboratory Test	Observed Value	Reference Range	Interpretation
Oxygen saturation (ABG)	80%	>95%	Decreased
PO₂ (ABG)	36 mmHg	80-100 mmHg	Decreased
Arterial pH (ABG)	7.44	7.35-7.45	Normal

Echocardiogram demonstrated severe PA/IVS, a severely hypoplastic tricuspid valve (Z-score: -3.7, annular diameter 6.4 mm), and a markedly hypertrophied, spongiform, and hypoplastic right ventricle. Multiple coronary sinusoids were visualized, with extensive ventriculocoronary connections and evidence of right ventricle-dependent coronary circulation. A secundum atrial septal defect measured 5 mm with right-to-left shunting. A tortuous patent ductus arteriosus (PDA) supplied blood to the branch pulmonary arteries, with reverse flow noted.

**Figure 1:**
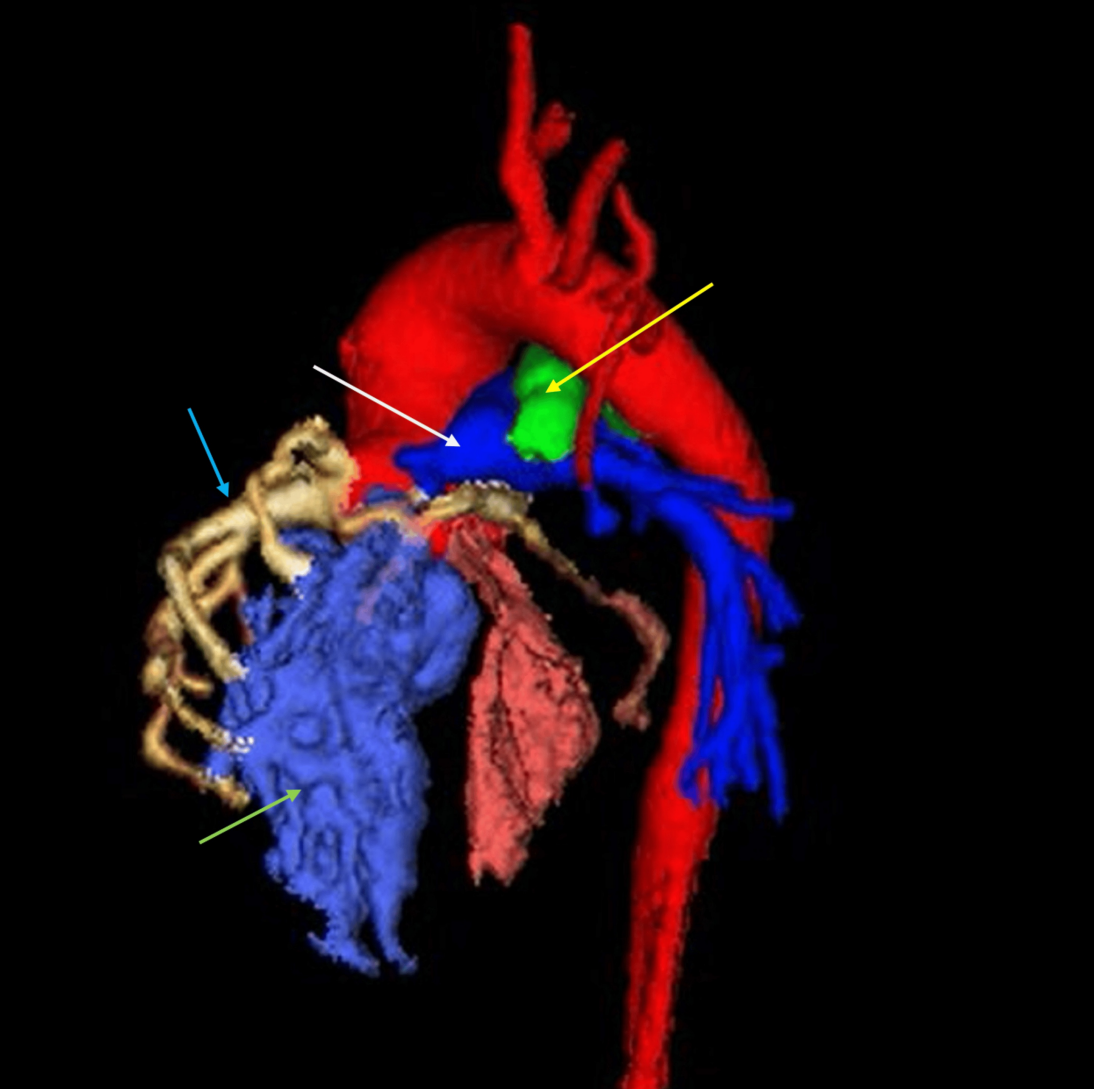
Three-dimensional reconstruction of a computed tomography image demonstrating markedly enlarged coronary arteries (blue arrow) communicating with the hypertrophied and hypoplastic right ventricle (green arrow), complete atresia of the pulmonary artery (white arrow), and an enlarged, tortuous patent ductus arteriosus (yellow arrow).

**Figure 2:**
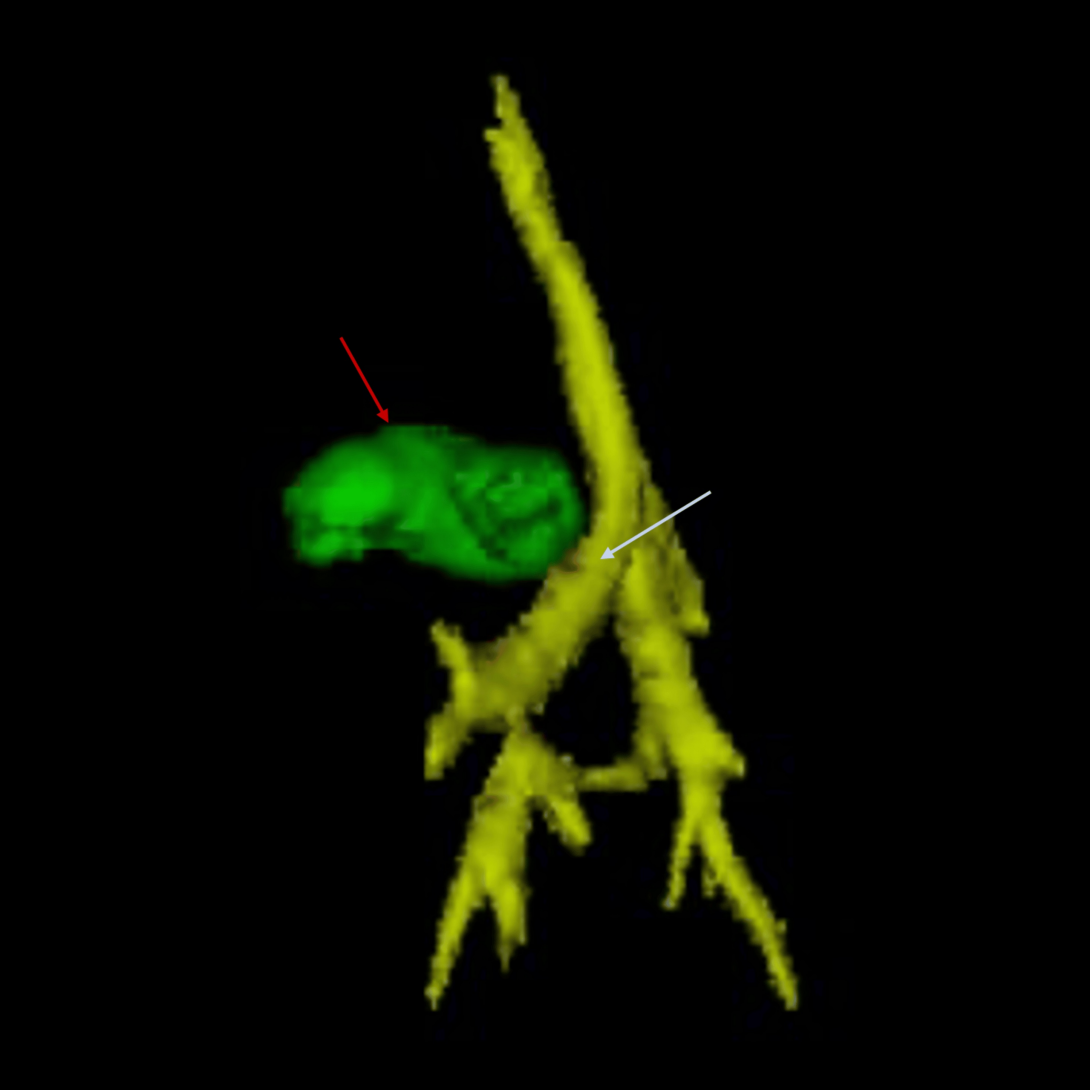
Three-dimensional reconstruction of a computed tomography image showing compression of the left main bronchus (white arrow) by an enlarged, tortuous patent ductus arteriosus (red arrow), resulting in airway obstruction.

**Figure 3:**
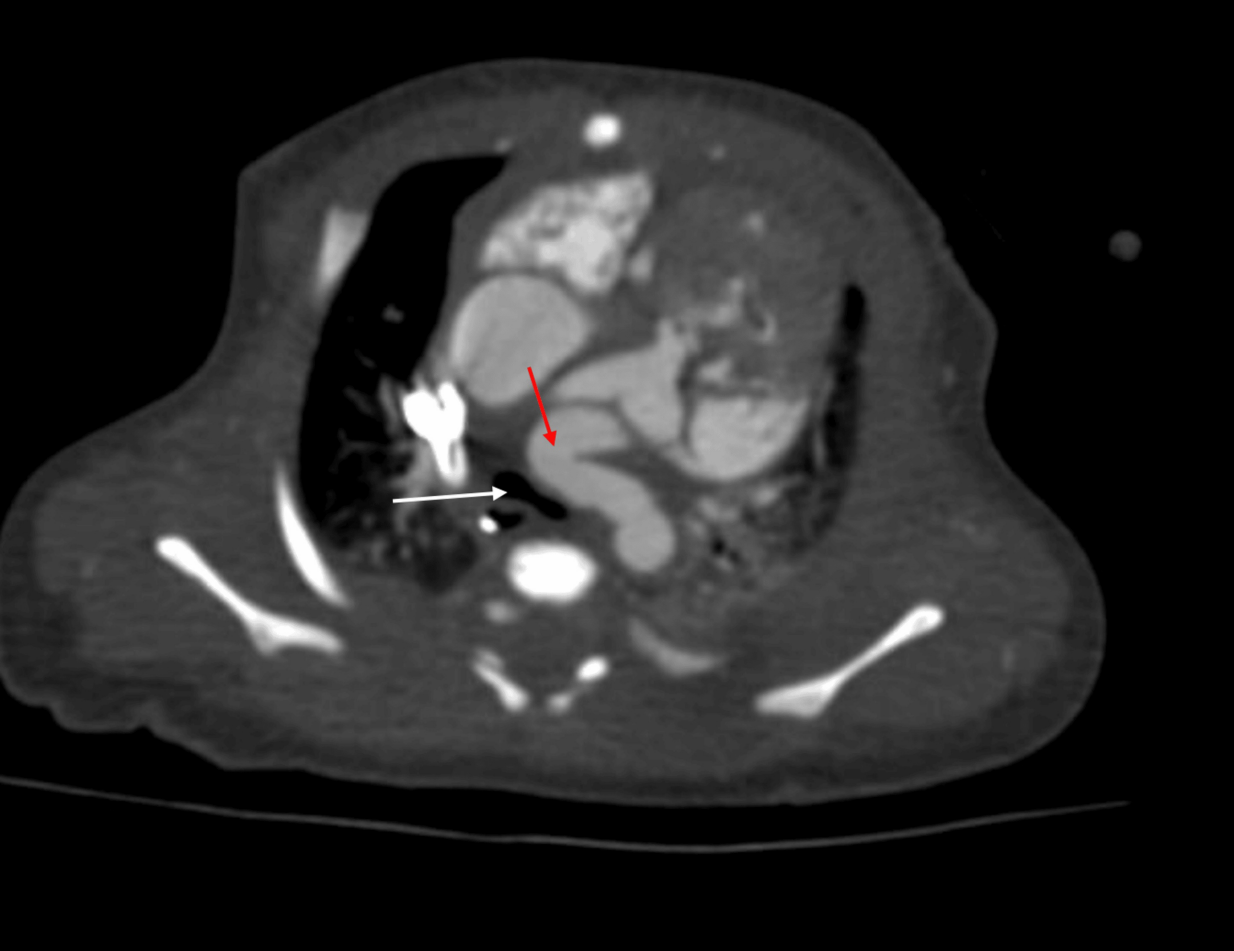
Axial computed tomography image of the enlarged, tortuous patent ductus arteriosus (red arrow) compressing the left main bronchus (white arrow).

**Figure 4:**
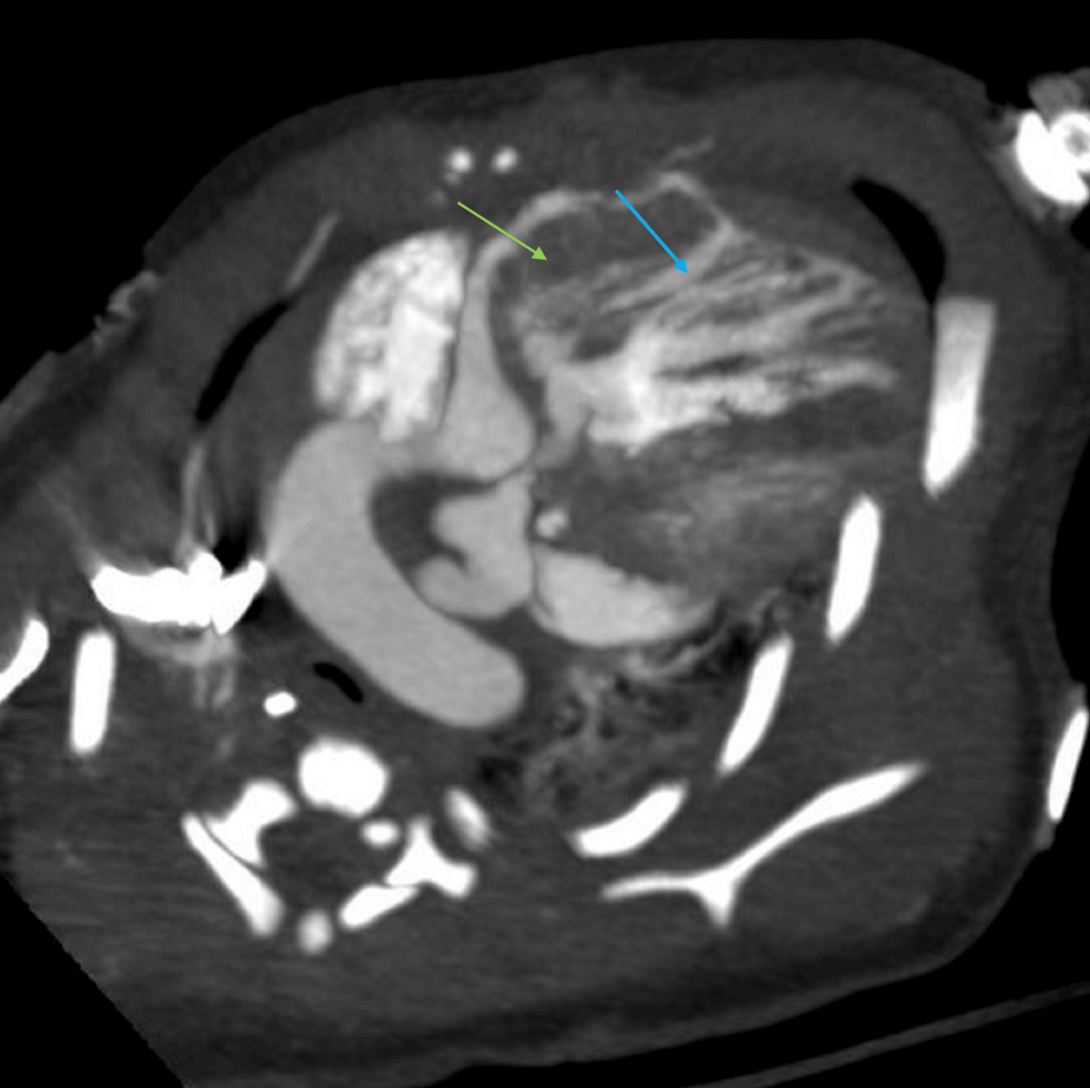
Axial computed tomography image of the markedly dilated coronary arteries (blue arrow) communicating with the right ventricle (green arrow).

Cardiac CT was obtained to further assess the coronary anatomy and vascular structures. CT confirmed complete pulmonary atresia, markedly dilated coronary arteries (right coronary artery measuring 4.4 mm and left coronary artery measuring 2.7 mm), and a large, tortuous PDA measuring 15 mm in length and 7.5 mm in diameter. Proximal coronary ostial atresia was excluded. The PDA caused posterior displacement and narrowing of the airway, contributing to left lung base compression. The right pulmonary artery measured 4.8 mm, the left measured 5.5 mm, and the main pulmonary artery measured 4.7 mm. These measurements were consistent with the echocardiographic findings and provided a comprehensive anatomical map of the pulmonary circulation (Figures 1, 2). Axial imaging further confirmed the coronary and airway findings, illustrating the extensive communication between the coronary arteries and right ventricle, and the compressive effects of the PDA on the left main bronchus (Figures 3, 4). The imaging findings were consistent with right ventricle-dependent coronary circulation, highlighting the high risk of myocardial ischemia if right ventricular decompression were to be attempted. Neither the echocardiogram nor cardiac CT revealed evidence of left ventricular dysfunction.

Following completion of the cardiac CT, the patient was successfully extubated to a low-flow nasal cannula at 1 L/min with 21% FiO₂. He maintained stable oxygen saturations ranging from the mid-70s to low-80s, consistent with his underlying ductal-dependent physiology. By the next day, he tolerated weaning to room air without signs of respiratory distress, confirming clinical stability prior to surgical intervention. Given the presence of coronary sinusoids and a severely hypertrophied and hypoplastic right ventricle, the multidisciplinary team determined that the patient was an appropriate candidate for single-ventricle palliation with a systemic-to-pulmonary shunt. This approach was favored over cardiac transplantation, as the coronary anatomy and ventricular physiology supported palliation as a feasible and lower-risk strategy.

## Discussion

This case illustrates the complex anatomy associated with PA/IVS with suspected right ventricle-dependent coronary circulation and emphasizes the complementary role of CT imaging in surgical planning and its unique ability to identify critical extracardiac findings such as airway compression. 

The following imaging findings were pivotal in confirming the diagnosis of PA/IVS with suspected right ventricle-dependent coronary circulation and highlight the complexity and uniqueness of this case, particularly the contributions of CT imaging in revealing both intracardiac and extracardiac anatomy. The first notable finding is the markedly enlarged coronary arteries, with multiple ventriculocoronary artery connections to the right ventricle. The proximal right coronary artery measured 4.4 mm, and the left measured 2.7 mm, compared to a normal diameter of approximately 1 mm in newborns [7]. The second unique finding is the extremely enlarged PDA, which measured 15 mm in length and 7.5 mm in diameter. Typically, a PDA diameter greater than 3 mm is classified as large; thus, this diameter significantly exceeds the normative understanding of a large PDA [8]. This enlarged PDA primarily caused compression of the left main bronchus and distal trachea, though concurrent mild contributions from the pulmonary artery and aortic arch cannot be excluded. The third finding is complete pulmonary atresia. The hypoplastic pulmonary artery measured only 4.7 mm in diameter, well below the normal range of 9-12 mm for full-term newborns [9]. 

Although echocardiography remains the first-line modality, CT offers superior spatial resolution and 3D visualization of the coronary arteries, pulmonary outflow, and great vessels. In similar congenital heart conditions, such as transposition of the great arteries or tetralogy of Fallot, accurate visualization of coronary artery anatomy using CT is essential to prevent inadvertent injury to anomalous coronary vessels during or following surgical intervention [10]. Importantly, CT provided unparalleled visualization in this case, not only of intracardiac and coronary anomalies but also of the unique extracardiac complication of airway compression due to the enlarged PDA, a rarely described finding in PA/IVS [10,11]. Given that the airway compromise appeared moderate on CT, the patient’s cyanosis was likely multifactorial, with contribution from impaired coronary perfusion from right ventricle-dependent coronary circulation. Notably, the patient’s oxygenation stabilized following extubation, with room air saturations maintained in the 70s-80s, suggesting that the degree of airway compression was not severe enough to necessitate prolonged mechanical ventilation. 

While most prior reports have focused on intracardiac findings and coronary assessment, airway compromise from ductal compression is seldom highlighted in the PA/IVS literature [4,6,10,11]. Although the developmental relationship between PA/IVS, enlarged ductal anatomy, and associated airway compression remains unclear, continued reporting of such complex cases may help clarify underlying mechanisms and inform future diagnostic and surgical approaches. This case underscores the importance of including airway evaluation in CT protocols for congenital heart disease, particularly when large ductal or vascular structures are present.

In PA/IVS, preoperative coronary assessment is critical, as surgical decompression of the right ventricle can trigger myocardial infarction when right ventricle-dependent coronary circulation is present [12]. Notably, CT extends beyond vascular evaluation; it can detect extracardiac complications, such as the airway compression seen in this case, which has major implications for perioperative management [6]. Advances in 3D reconstruction and printing have further enhanced CT’s utility, allowing surgeons to preoperatively simulate and plan complex interventions [13].

This case exemplifies the importance of integrating detailed anatomical assessment, both coronary and extracardiac, into early surgical decision-making. CT played a pivotal role by confirming the absence of coronary ostial atresia and revealing severe airway compromise, informing the selection of a surgical rather than catheter-based approach. Such individualized evaluation is crucial in determining suitability for single-ventricle palliation or transplant consideration [14]. This case highlights CT’s unique contribution in diagnosing rare, clinically significant findings that would likely remain undetected with echocardiography alone.

While catheter-based interventions, such as ductal stenting, have become more common in PA/IVS management, the complex coronary and airway anatomy in this case favored a surgical approach [2].

## Conclusions

In neonates with PA/IVS and suspected right ventricle-dependent coronary circulation, CT imaging offers detailed anatomical information critical for surgical decision-making. This case illustrates how CT can supplement echocardiographic findings by clarifying coronary anatomy, evaluating airway involvement, and guiding palliative strategies in patients with complex congenital heart disease. Importantly, this case emphasizes the added value of cross-sectional imaging in identifying extracardiac complications, such as airway compression, which may significantly influence perioperative planning and long-term outcomes. As congenital heart disease management continues to evolve, early multimodal imaging remains essential in tailoring individualized, anatomy-driven approaches to care.
